# HUMERAL SHAFT NONUNION. RETROSPECTIVE STUDY OF SEVERAL SURGICAL TECHNIQUES

**DOI:** 10.1590/1413-785220263401e296589

**Published:** 2026-02-13

**Authors:** Victor Nagib Queiroz Balech, Rodrigo Emmanuel Leimig Telles Parente, Enzo Baldas Skuk, Ralph Walter Christian, José Octavio Soares Hungria, Caio Zamboni

**Affiliations:** 1 Santa Casa de Misericordia de Sao Paulo, Grupo do Trauma, Sao Paulo, SP, Brazil.

**Keywords:** Pseudoarthrosis, Humeral Fracture, Fracture Healing, Pseudoartrose, Fratura do Úmero, Consolidação da Fratura

## Abstract

**Objective:**

To evaluate demographic aspects, risk factors, trauma mechanisms, and therapeutic outcomes in humeral shaft nonunion.

**Methods:**

Retrospective analyzing of 14 patients treated between 2011 and 2024 at a quaternary hospital. Evaluating Demographic data, fracture characteristics, treatments, and outcomes were descriptively assessed.

**Results:**

The average patient's age was 51 years. Half of the patients were involved in domestic accidents, and half in traffic accidents. 58% of fractures occurred in the middle third of the shaft, and 93% were classified as atrophic nonunions. Healing failure was linked to lack of bone contact, implant loosening, and infection. After definitive treatment, 71% achieved union, 21% remained in nonunion. Bone grafting combined with compression was associated with superior outcomes.

**Conclusion:**

Trauma mechanism, AO classification, and comorbidities were not significant predictors of healing. Grafting associated with compression was crucial for union achievement. ***Level of Evidence IV; Case series.***

## RESUMO

**Objetivo:**

Avaliar aspectos demográficos, fatores de risco, mecanismos de trauma e desfechos terapêuticos na pseudoartrose da diáfise do úmero (PDU).

**Métodos:**

Análise retrospectiva de 14 pacientes tratados entre 2011 e 2024 em hospital quaternário, com análise descritiva de dados demográficos, características das fraturas, tratamentos e desfechos.

**Resultados:**

A média de idade dos pacientes foi de 51 anos. Metade dos casos sofreu acidentes domésticos, e a outra metade, acidentes automobilísticos. 58% das fraturas ocorreram no terço médio da diáfise, com predominância de pseudoartrose atrófica (93%). Falhas de consolidação foram associadas à infecção, ausência de contato ósseo e soltura de material. Após o tratamento definitivo, 71% consolidaram, 21% permaneceram em pseudoartrose. A utilização de enxerto ósseo e compressão demonstrou melhores resultados.

**Conclusão:**

Mecanismo de trauma, classificação AO e comorbidades não foram fatores preditivos significativos. A combinação de enxertia óssea e compressão demonstrou bons resultados na consolidação. ***Nível de Evidência IV; Série de casos.***

## INTRODUCTION

Humer diaphysis fractures comprise 1% of all fractures, and the incidence of humeral shaft nonunion (HSN) remains low. According to the French Agency of Information on Hospital Care (ATIH), 8% of diaphysis fractures evolved to non-union.^1^

Sarmiento reported consolidation rates above 95% for humeral shaft fractures treated non-operatively; however, more recent authors have not achieved the same effectiveness with non-operative treatment.^2^ Toivanen et al., in 2005, reported a nonunion rate of 22.5% in non-surgical management, mainly associated with fractures in the proximal segment.^3^ Rutgers and Ring, in 2006, had failure in 10% of their patients treated non-operatively, also more related—similarly to Toivanen et al.—to fractures in the proximal segment of the humeral shaft.^4^ The indications for surgical treatment have been expanding, being absolute (open fracture associated with nerve injury, polytrauma, association with articular fracture, fractures in pathological bone, floating elbow, and failure of non-operative treatment) and relative (associated obesity, muscular atrophy, bilateral fracture, and association with brachial plexus injury).^5^ However, between operative and non-operative approaches for humeral shaft fractures, nonunion has remained a complication.

According to Gonçalves et al., pseudoarthrosis reached 10.3% of patients treated with open reduction and internal fixation, the method still considered today the most efficient, said gold standard.^6^ Thus, it becomes necessary to study the complications arising from the various therapies used in cases of diaphyseal fractures of the humerus. Another complication associated with poor prognosis of humeral shaft nonunion (HSN) is infected pseudoarthrosis, which in turn has risk factors such as high-energy trauma with exposure, advanced age, extensive soft tissue loss, immunodeficiency, diabetes, smoking, vascular insufficiency, and prolonged hospital stay, among others.^7^

## OBJECTIVE

Analyze possible demographic factors, risk factors, trauma mechanisms that led to HSN and how the procedures carried out led to various outcomes in the treatment of the disease.

## METHOD

The data used were from medical records, radiographs and tomographs from the hospital's files. Demographic characteristics of patients were collected: age, gender, location of fracture trace, dominance, comorbidities, classification of trauma mechanism, laterality, initial treatment and definitive treatment. In addition, the date of the fracture, the initial approach, the date of the final approach, the use or not of the graft, methods, principles of stability used and time between the approaches were analyzed. The data was analyzed descriptively. We used Weber and Cech's radiographic classification developed in 1976 that divides pseudoarthrosis into hypertrophic, oligotrophic and atrophic.^8^

The terms of consent were signed by the patients we gained access to; however, the same process was not carried out for those whose contact was not effective. The waiver request was requested and approved by the Ethics Committee (CAAE: 83200924.0.0000.5479). From January 2011 to July 2024, we analyzed 17 patients who had HSN in a quarterly health care institution.

The inclusion criteria are pseudoarthrosis of membrane fracture of the homer (defined in this study as lack of evidence of union after a minimum period of six months) and maintenance of outpatient follow-up. We use the AO classification that defines the diaphysis as the part of the bone that is located between the distal and proximal extremities that is limited by the Heim square. Therefore, cases that did not fall within these were excluded.^9^

Patients with incomplete data, poor quality X-rays, patients with consolidation subsequently classified as retarded and not as pseudoarthrosis and those who did not agree to participate in the study were excluded. There were 14 patients who met the exclusion and inclusion criteria.

The patients analyzed had an average age of 51 years (26 - 77), of the 14 patients analyzed seven are men. Comorbidities were found in six (43%) patients. Among the comorbidities, three patients are smokers, three are hypertensive, and one has heart disease. All patients were right-handed, ten (72%) suffered fractures in the non-dominant limb.

## RESULTS

The trauma mechanisms observed in the treated patients seven (50%) suffered car accidents and seven (50%) domestic accidents (fall from stairs, fall to the ground). Of the car accidents, six evolved to consolidation after the treatment of pseudoarthrosis and one remained in pseudoarthrosis. In the domestic accidents four consolidated, one was performed endoprosthesis and two continued in pseudoarthrosis.

Regarding the fracture location, eight (58%) were in the middle third, four (28%) in the proximal third, and two (14%) in the distal third of the diaphysis. The fracture types were as follows: five oblique, one spiral, seven transverse, and two comminuted.

In the AO classification of fractures, among the patients analyzed, three 12 A1, five 12 A2, two 12 A3, two 12 C2, two 12 C3. (Table 1) In the initial approach, inadequate treatment with a bone clamp was performed in six (43%) of the patients, two (14%) patients performed bridge plate (one by the technique of Belangeiro and Livani, a blocked plate), two (14%) patients used compression plate and traction screw, three (22%) performed compression plate, one (7%) prosthesis and double orthogonal plate.

**Table 1:** Results.

	AO Classification	Type of pseudoarthrosis	Final treatment	Use of Graft	Consolidation at the end of treatment
1	12 A2	Atrophic	Neutralization plate and traction screw	Y	Y
2	12 A2	Atrophic	Treatment of infection	N	N
3	12 A2	Atrophic	Compression plate	Y	N
4	12 C2	Atrophic	Compression plate	Y	N
5	12 A2	Hypertrophic	Compression plate	N	Y
6	12 C3	Atrophic	Neutralization plate and traction screw	Y	Y
7	12 C2	Atrophic	Compression plate	Y	Y
8	12 A3	Atrophic	Compression plate	Y	Y
9	12 A3	Atrophic	Compression plate	Y	Y
10	12 A2	Atrophic	Compression plate	Y	Y
11	12 A1	Atrophic	Treatment of infection	N	Y
12	12 C3	Atrophic	Endoprosthesis	--	--
13	12 A1	Atrophic	Plate in wave	Y	Y
14	12 A1	Atrophic	Compression plate	N	Y

Legend: Y-Yes; N-No. **Source:** Database of patients of orthopedics and traumatology of the hospital Santa Casa de Misericórdia de São Paulo.

Of the 14 patients analyzed, 13 were considered with pseudoarthrosis atrophic and one with hypertrophic. In nine (64%) there was a need to use a graft in the final treatment.

Among the treatments for pseudoarthrosis, one opted for compression board and shortening more traction screw in two (14%), compression board in eight (58%), infection treatment in two (14%) and endoprosthesis one (7%), wave plate in one (7%).

The radiographic factors observed in cases of humeral diaphysis fractures that progressed to pseudoarthrosis were lack of fragment contact in seven (50%) patients, loosening of fixation material in four (28%), and fracture-related infection in three (22%).

Of the patients analyzed after follow-up time, ten (71%) showed consolidation, three (21%) remained in pseudoarthrosis and in one patient (7%) there was a need to perform endoprosthesis. After the surgical procedure two 12 A2, a 12 C2 maintain the outcome of pseudoarthrosis. A 12 C3 endoprosthesis was performed.

## DISCUSSION

Despite the therapeutic possibilities, there is no consensus or precise protocols on the best strategy to be adopted in the context of pseudoarthrosis of the humerus.^10^

In disagreement with Raven et al. we were unable to correlate high energy as a risk factor for HSN and neither the mechanism of trauma nor the severity of the fracture taking as reference the AO classification proved relevant in our patients.^7^

Peters et al. in a systematic review with metanalysis concluded that the open reduction and fixation with plates and screws associated with the autologous graft achieved higher consolidation rates and a small rate of complications; similarly, the external fixation also presented good bond rates, in addition to presenting an advantage when the patient presented infection according to the authors.^11^ In eight (57%) of the 14 patients, we achieved increased stability with plates and screws as a definitive approach and 71% (ten) of these treatments with focus compression either by compression by the plate or by neutralization plate associated with traction infusion showed consolidation.

Fozzato et al. concluded that, in addition to the open reduction and internal fixation with plates and screws in a stable way associated with corticospongiform graft, the complete removal of non-viable tissue from the pseudoarthrosis focus led to higher ratio of union.^12^ In this context, we performed this revitalization with insertion in seven patients, of which five showed consolidation. Healy et al. recommend the ingestion as a routine, and should not be reserved only for cases of atrophic pseudoarthrosis.^13^ Despite little mention, we found that the acute shortening with compression could also help revitalize, exposing and coupling viable edges with better quality bone. After resection of the devitalized tissues, this shortening was performed in nine patients.

We observed that the stable fixation with plates and screws presented good results, however Patel et al. demonstrated that another method – Ilizarov's circular external fixator – also presented high consolidation rates, having achieved union in 15 of 16 patients.^14^ In our case, in a patient with infection already under control, treatment with linear fixator was attempted, unsuccessfully and with reabsorption of the graft. We only obtained consolidation when we exchanged the fixer for revitalization, shortening, grafting and compression with plate and screws. A second patient was also treated with a monoplanar external fixator for infection control. Although the bacterial infection was resolved with cleaning, debridement, and placement of antibiotic-loaded cement, bone consolidation was still not achieved. Despite the pseudoarthrosis and functional limitations, the patient declined further surgery. Our experience suggests that the use of a monoplanar external fixator is effective for infection control when combined with cleaning, debridement, and placement of antibiotic cement, but bone consolidation was not achieved in either of the two patients.

**Figure 1:**
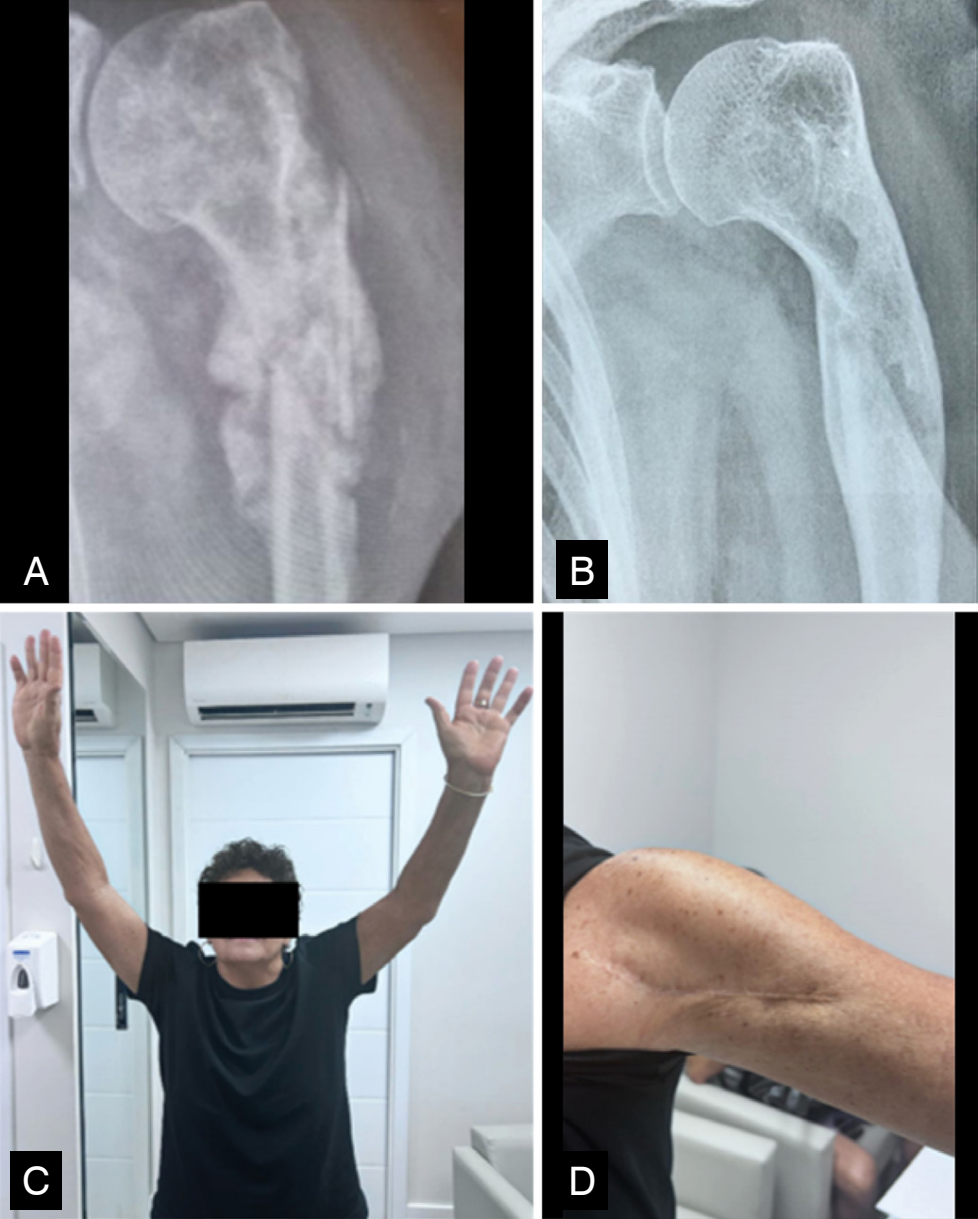
X-rays of patient 11 showing infected hypertrophic pseudoarthrosis (A) and consolidation (B) after treatment of infection; and clinical images of functionality (C) and scar (D).

Source: Personal file of the authors, with informed consent and anonymity guaranteed.

Although most studies indicate that the main modifier of the natural history of pseudoarthrosis is the gain of stability and osteogenic stimulation, we observed that, in the context of infected pseudoarthrosis, one of our patients presented consolidation of the fracture while treating the infection, even after removal of synthesis material. This patient did not use the fixator because the fracture was in the proximal diaphysis region. Opted for the time by removing the plate and screws, cleaning, revitalizing and placing cement with local antibiotic. The proximal region was preserved considering the next fixation procedure. However, after four weeks, the cement with antibiotic was removed and the fracture was consolidated, not requiring further fixation. (Figure 1)

**Figure 2:**
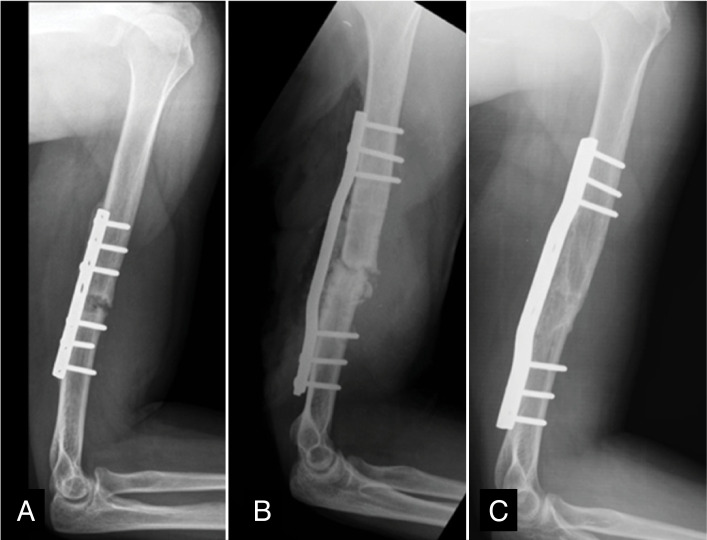
X-rays of the patient 13 showing atrophic pseudoarthrosis with reabsorption of the fracture focus (A), after graft placement and fixation with wave-molded plaque (B) and after bone consolidation (C).

Source: Xero Viewer Data Base from the Santa Casa de Misericórdia de São Paulo.

In one of the patients who presented with atrophic HSN, we used RAFI with a long plate, wave-shaped with graft. The reason was that there was great bone loss in the screw holes due to the release of the implant associated with non-consolidation, being enlarged the working area with a long plate protecting these holes and being fixed in viable bone, which was wave-shaped for placement of tricortical graft under the implant and spongy in the cortical *trans*, leading to the consolidation of pseudoarthrosis. (Figure 2)

**Figure 3:**
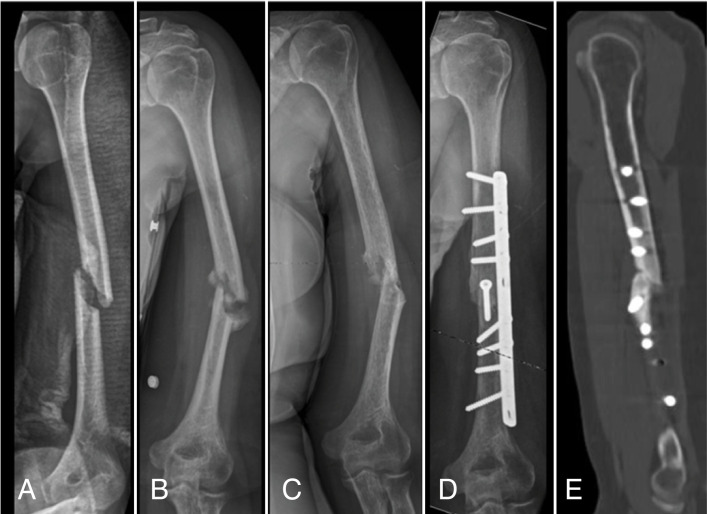
Images of the patient 1. X-rays showing the initial aspect of the fracture, evolution of 17 months, 29 months, 41 months (A, B, C and D); and tomography after 41 months of the fracture (E).

Source: Xero Viewer Data Base from the Santa Casa de Misericórdia de São Paulo.

Of our 14 patients, 13 presented atrophic HSN; of the patients with atrophic pseudoarthrosis, three initially presented characteristics interpreted by us as an attempt to form bone calo, initially classified as hypertrophic pseudoarthrosis, however, in the follow-up, this tissue was reabsorbed, becoming considered pseudoarthrosis avascular. These three were under 60 years old and had fragmented fractures. Knowing that these patients initially classified as hypertrophic pseudoarthrosis evolved into atrophic pseudoarthrosis, we decided to recommend ingestion for all cases of pseudoarthrosis. (Figure 3)

The patient who needed to undergo bone replacement by a total endoprosthesis of the humerus had two focuses of pseudoarthrosis in the diaphysis despite several surgeries. There was rupture and release of the implants. As an antecedent, she had rheumatoid arthritis and had done a complete prosthesis of the ipsilateral shoulder more than ten years ago.

**Figure 4:**
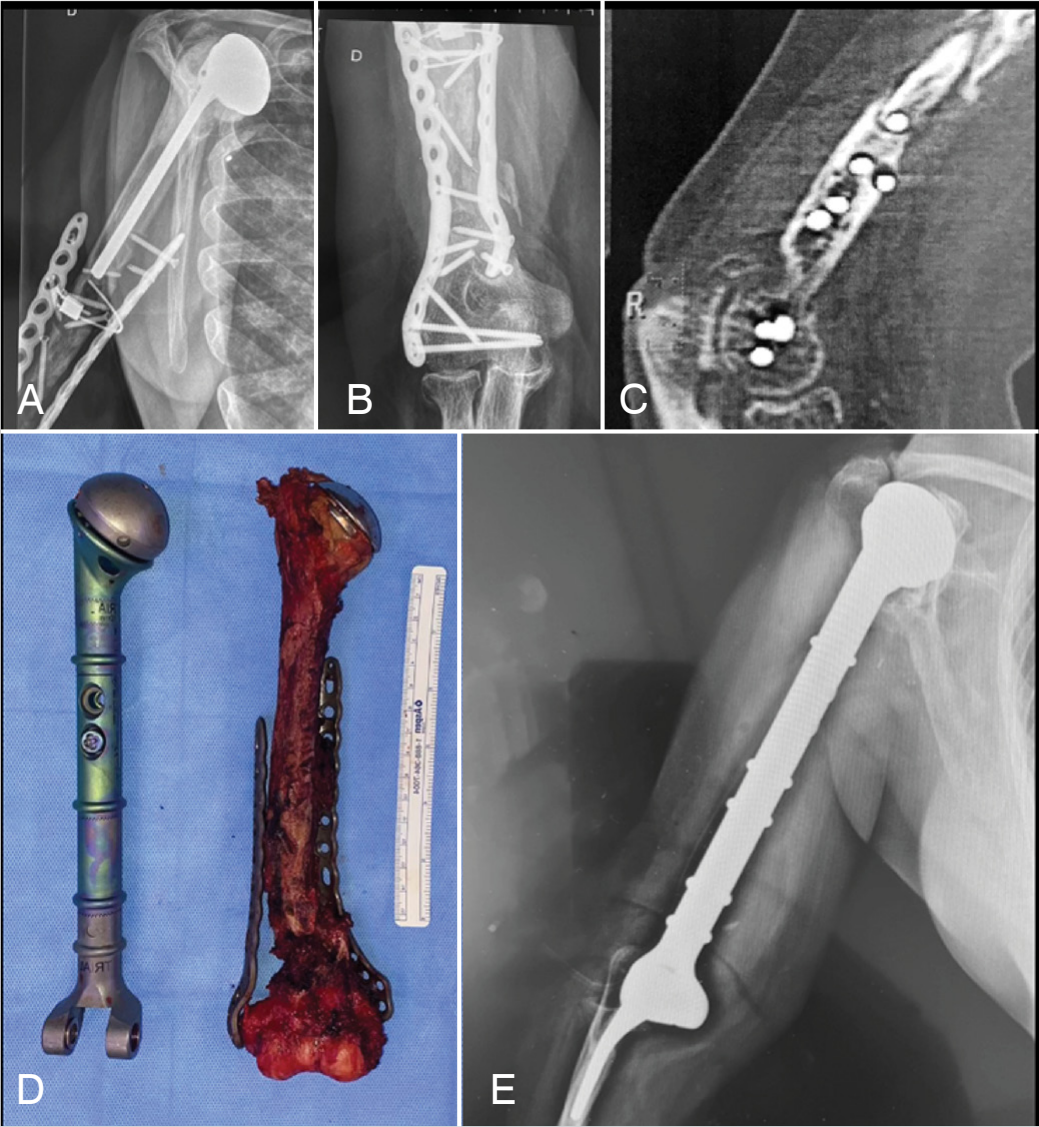
Images of the patient 12. X-rays and tomography showing the two focuses of pseudoarthrosis, fracture and release of the implant and the complete prosthesis of the shoulder (A, B and C). Image of the resected humerus and evidence of the endoprosthesis model used (D). Final X-ray with total replacement of humerus (E).

Source: Personal file of the authors, with informed consent and anonymity guaranteed.

One of the limiting factors was that patients had irregular follow-up and a temporary standardization was not possible. (Figure 4)

## CONCLUSION

Mechanism of trauma, initial AO classification of the fracture and comorbidities were not significant for consolidation of patients in this series of cases. The best results were obtained from the use of the graft and compression of the pseudoarthrosis focus with or without shortening.

## Data Availability

The contents underlying the research are available in the manuscript.
